# Computer guided resection and reconstruction of intra-osseous zygomatic hemangioma: Case report and systematic review of literature

**DOI:** 10.1016/j.ijscr.2019.12.015

**Published:** 2019-12-17

**Authors:** Ahmed Talaat Temerek, Sherif Ali, Mohamed Farid Shehab

**Affiliations:** a Oral and Maxillofacial Surgery Department, Faculty of Dentistry, South Valley University, Qena, Egypt; b Oral and Maxillofacial Surgery Department, Faculty of Dentistry, Cairo University, Cairo, Egypt

**Keywords:** Vascular malformations, Hemangioma, Zygomatic bone, Computer-assisted surgery, Reconstructive surgery

## Abstract

**Objectives:**

to systemically integrate the available data on various published treatment strategies for intraosseous hemangioma of the zygoma in an updated comprehensive systematic review, and to present a new case of treated resection and immediate reconstruction using computer guided patient specific composite graft.

**Case presentation:**

A 29 years old female patient with intraosseos zygomatic hemangioma treated using computer guided resection and immediate reconstruction. The resection was performed using a resection template constructed based on preoperative 3D planning. The reconstruction was performed using ramus bone graft and titanium mesh. The patient was followed up for 3 years with no complications.

**Methods:**

An electronic search was performed on 3 data bases; additional hand search of bibliographies of selected articles was performed. Eligibility criteria include human studies presenting intraosseous zygomatico-orbital hemangioma with clearly reported therapeutic strategies.

**Results:**

53 articles with 73 cases were included. The lesion was highly prevalent in females compared to males (2.28:1). Fifth decade represents the highest prevalence with mean age of 44.1 ± 1.8 years. The main patient concern was swelling and facial deformity. Total tumor resection can assure no recurrence, while partial resection and curettage are associated with high recurrence rate.

## Highlights

• Intraosseous zygomatic hemangioma is highly prevalent in females compared to males (2.28:1), with mean age of 44.1 ± 1.8 years.
• The main patient concern in intraosseous zygomatic hemangioma is swelling and facial deformity.
• Partial resection and curettage are associated with high recurrence rate.
• Total tumor resection can assure no recurrence proved for over 10 years of follow-up, with minimal intraoperative bleeding occurred in most of the cases.
• Computer guided surgery for resection and reconstruction of intraosseous zygomatic hemangioma facilitates the surgical procedures.

## 1 Introduction

In 1982 Mulliken and Glowacki described 2 types of vascular anomalies: Infantile Hemangioma and Vascular Malformations (VMs). Then, by 1996 it was categorized by the International Society for the Study of Vascular Anomalies (ISSVA) into: “tumors” including hemangioma, hemangioendotheliomas, angiosarcoma, miscellaneous; and “malformations” including slow flow malformations, high flow malformations. VMs are thought to be developmental lesions that arise from lymphatic or blood vessels and can occur in soft or hard tissue [1,2]. Intra-osseous vascular anomalies are uncommon neoplasms accounting for less than 1 % of all osseous neoplasms. In craniofacial region, the highest incidence was reported in parietal bone followed by the mandible [3].

Although it’s rare incidence in zygomatic bone, it represents a true dilemma. The zygomatic bone is considered to be a keystone in facial aesthetics; it provides facial symmetry, supports the eye, and contributes to overall facial contour [4]. Preoperative awareness of the vascular nature of a zygomatic lesion is crucial. Plain x-rays, CT scan, magnetic resonance imaging (MRI), ultrasound and carotid aniography are considered to be useful tools in preoperative preparation and diagnosis [5]. Treatment options included surgical interference in the form of total tumor resection through narrow safety margin with or without reconstruction. Also, non-surgical modality in the form of follow-up after confirming the diagnosis was reported [6]. Reconstruction of the residual zygomatic defects poses a challenge to the surgeons to provide the best esthetic results with a wide variety of treatment options including autogenous bone grafts harvested from iliac crest, rib, and calvaria. Alloplastic materials, titanium meshes and patient specific implants (PEEK) have also been utilized especially with the evolution of computer assisted surgical techniques and 3D printing which augments the preoperative surgical planning and diagnosis [7].

In this study, we aim to present a case of intraosseous zygomatic hemangioma treated by resection and immediate reconstruction using a novel method employing a computer guided patient specific composite graft, and to systemically integrate the available data on various published treatment strategies for hemangiomas of the zygoma in an updated comprehensive systematic review. This case report was prepared following the SCARE 2018 criteria [8].

## 2 Case presentation

### 2.1 Patient information

A 29 years old female patient presented to our institution at February 2016 complaining of a slowly growing swelling of the right orbit causing facial disfigurement in the last 6 months, with no history of trauma incidence. The patient gave a history of intermittent throbbing pain together with multiple attacks of conjunctivitis and epiphora in the last 3 months. Her past medical history was non-significant. Family history revealed that no other family member had similar condition.

### 2.2 Clinical findings and diagnostic assessment

**Fig. 1:**
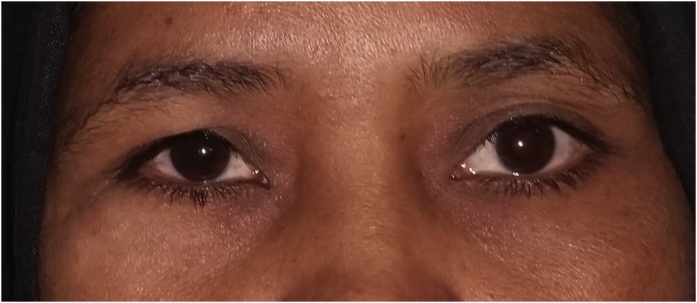
Clinical photo before surgery.

**Fig. 2:**
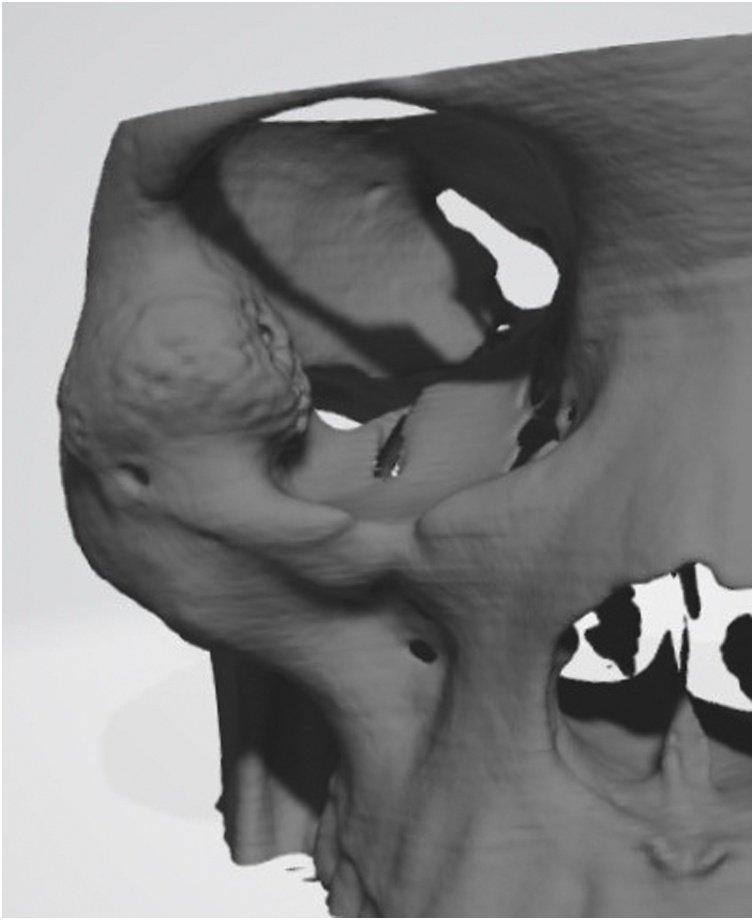
Radiographic picture showing the mass involving the right orbital floor, lateral wall, inferior and lateral rims plus anterior surface of zygomatic body.

Clinical examination revealed a hard, tender, immobile and palpable mass occupying the inferior and lateral orbital rims together with the malar surface of zygomatic bone (Fig. 1). Computed tomography (CT) showed a multilocular trabeculated, round, well-defined expansile bony lesion measuring 1.8 × 1,7 × 1,5 cm involving the right orbital floor, lateral wall, inferior and lateral rims plus anterior surface of zygomatic body (Fig. 2). MRI examination was requested for further evaluation, T1 and T2 weighted images showed intermediate signal intensity, with no associated soft tissue lesion. Areas of no signal were noticed that corresponded to the trabeculae seen on the CT study. Based on CT and MRI findings a preliminary diagnosis of intra-bony hemangioma was set. Needle aspiration was not decisive. Under local anesthesia an incisional bone biopsy using a trephine bur through a stab incision at the place of subciliary incision over the malar bone was done, minimal bleeding occurred and was controlled by direct pressure over the incision. The histopathological examination reported a cavernous hemangioma. Based on case history, clinical and radiographic findings the patient was informed with the treatment plan to resect and reconstruct the tumor using ramus bone graft, but due to cultural reasons she denied surgical intervention and asked for a monthly follow-up schedule. Six months later, a new CT revealed increase in lesion size reaching 2.3 × 2 × 1,7 cm. Moreover, she reported an increased incidence of throbbing pain, attacks of conjunctivitis and epiphora. At 9 months follow-up visit she asked for surgical intervention to remove the lesion.

### 2.3 Interventions

**Fig. 3:**
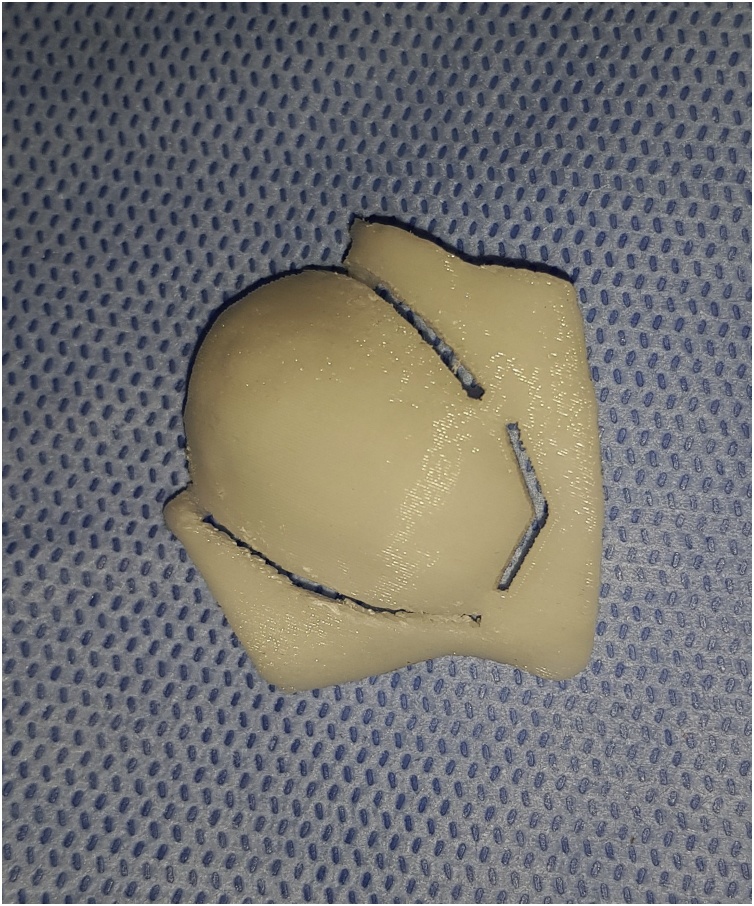
The surgical resection template.

**Fig. 4:**
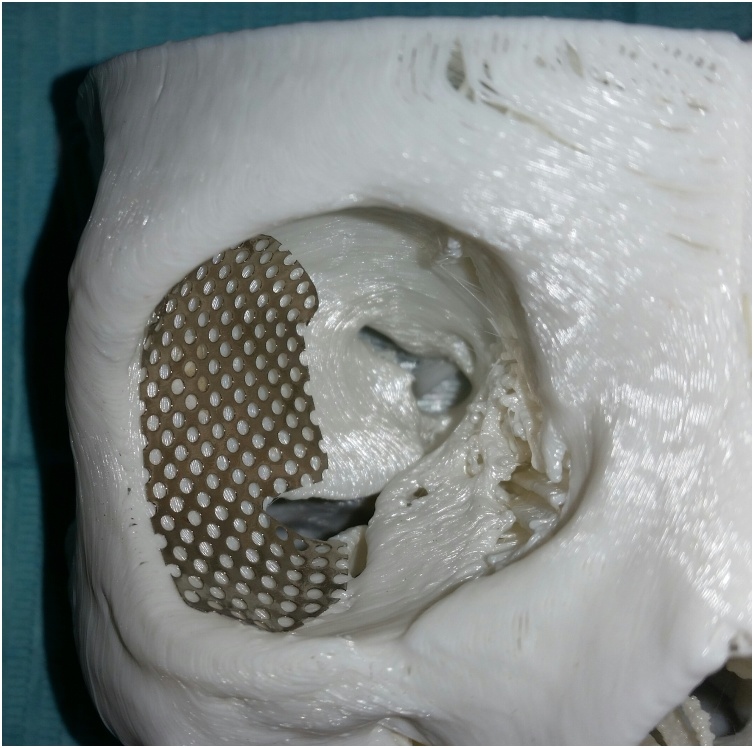
Titanium mesh adapted on the stereolithographic model constructed after virtual mirror imaging the orbito-zygomatic complex part of the normal side.

The CT scan was imported as DICOM files into surgical simulation software (mimics 19, Materialise) to provide better visualization and treatment planning. A 3D virtual model was constructed on the software for better identification and study of the tumor margin, planning of the surgical procedures. The lesion was identified on the virtual 3D model and the resection margins were set to create a surgical resection template (Fig. 3). The lesion was virtually removed and after studying the size, location and relations of the defect, a decision was made to recreate the lateral and inferior orbital rims plus anterior wall of the malar bone through an autogenous bone graft from the ipsilateral mandibular external oblique ridge, anterior border and lateral shelf of the ramus. Also, the orbital floor and lateral wall will be restored using a titanium mesh. A stereolithographic model together with the resection template were sent for 3D printing after virtual mirror imaging the orbito-zygomatic complex part of the normal side to occupy the diseased part. Upon this model trimming and prebending of the titanium mesh was done for restoring the patient orbital floor and lateral wall (Fig. 4).

**Fig. 5:**
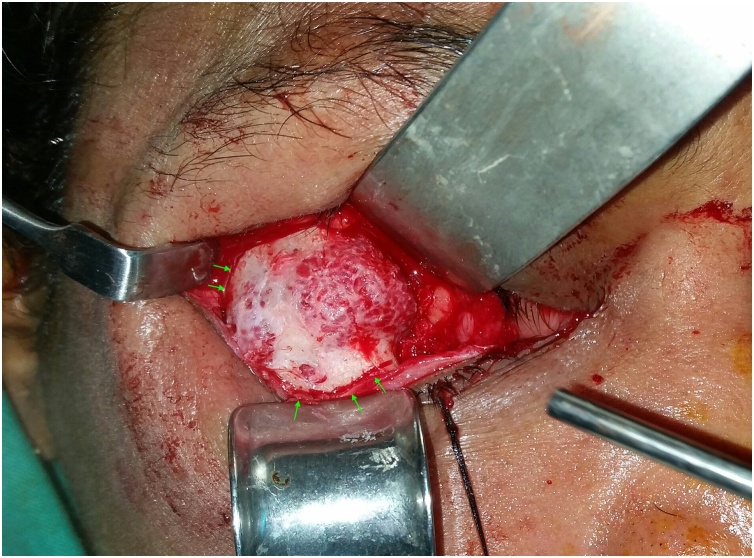
The resection margins marked according to the surgical template.

**Fig. 6:**
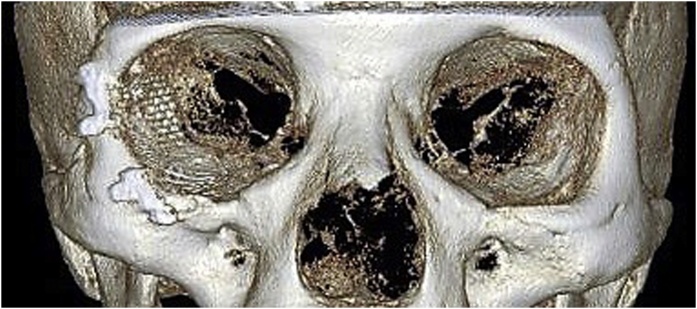
Postoperative CT showing the graft and titanium mesh probably adapted.

**Fig. 7:**
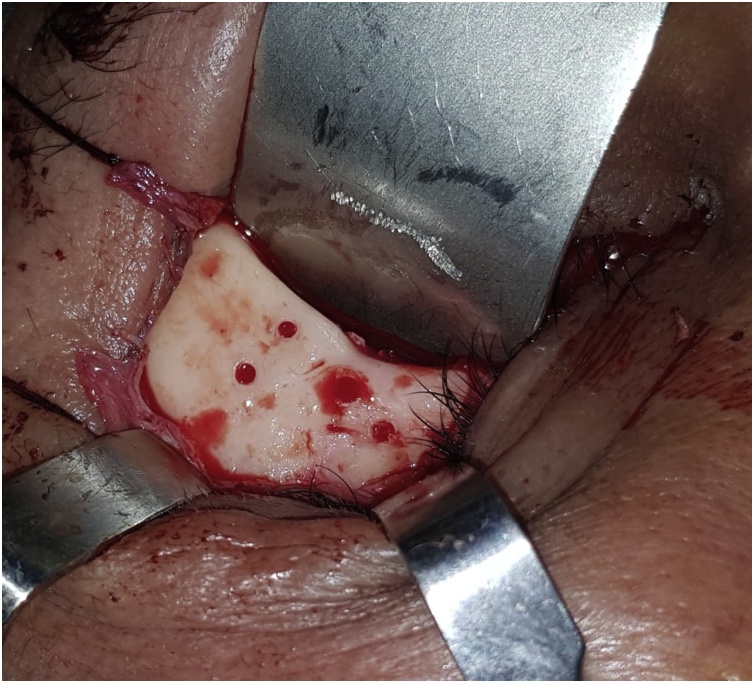
Ramal bone graft consolidation after removing of the hardware.

The surgical procedure included a transconjuctival approach with lateral canthotomy to expose the tumor mass. After exposure, the resection template was placed on the bone and the resection margins were marked, then the tumor was resected using a surgical micro-saw followed by curettage (Fig. 5). A vestibular incision was done to harvest the ramus bone graft using piezosurgery. The bone graft was fitted to the defect and fixed using titanium bone plates and screws. Orbital wall and floor were restored using a pre-contoured titanium mesh that was fixed using 2 titanium micro screws (Fig. 6). After 2 years, a second operation was done to remove the hard ware as the patient complained of a palpable plate where the bone graft showed good consolidation into the bony bed (Fig. 7)

### 2.4 Follow up and outcomes

**Fig. 8:**
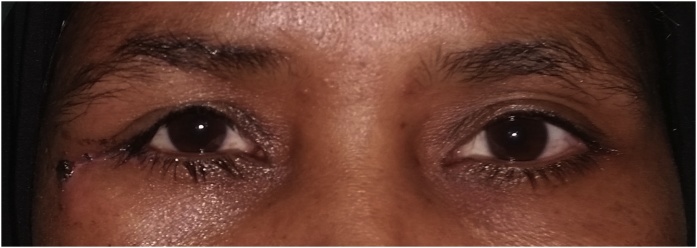
Clinical photo after surgery.

Regular follow-up every 6 m till 3 years postoperatively showed preserved ocular position vertically and horizontally, achievement of bilaterally symmetrical contour of the midface with no deformity, good consolidation of the bone graft into bony bed plus no recurrence as documented from the radiographic and clinical follow-up records (Fig. 8).

## 3 Methods

This review was performed in accordance with the preferred reporting items for systematic review and meta-analyses (PRISMA statement).

### 3.1 Eligibility criteria

We reviewed all clinical trials reporting intraosseous zygomatico-orbital hemangioma and discussing different treatment strategies. Studies were selected according the following criteria: Human studies reporting intraosseous zygomatico-orbital hemangioma (zygomatic bone, lateral & infraorbital rim); therapeutic strategies are clearly reported by the authors; Studies published in English. No limitation was placed regarding the study design, number of patients, and publication year. Cohort, case-control, cross-sectional, and case series studies including different facial hemangiomas were excluded if no sufficient data can be extracted regarding hemangioma of zygoma cases.

### 3.2 Search strategy

An electronic search was performed on 3 databases: The National Library of Medicine, Washington, DC (MEDLINE/PubMed), The Cochrane Central Register of Controlled Trials (CENTRAL); LILACS. The last electronic search was performed on 12th May 2018. Furthermore, hand search was performed on the major journals in the field of oral and maxillofacial surgery, from 2000 to 2018 (International Journal of Oral and Maxillofacial Surgery, Journal of Oral and Maxillofacial Surgery, Journal Of Cranio-Maxillofacial Surgery, British Journal of Oral and Maxillofacial Surgery, Oral Surgery Oral Medicine,Oral Pathology Oral Radiology, International Journal Of Oral Science, Journal Of The American Dental Association, Bmc Oral Health). Finally, the bibliographies of all articles selected for full-text screening were searched for relevant studies. The following search terms were used to search different databases: (“Hemangioma” OR “Vascular tissue neoplasm” OR “Vascular tissue malformation” OR “Primary intraosseous vascular malformation”) AND (“Zygoma” OR “Zygomatic” OR “Orbit” OR “Orbital” OR “Maxilla” OR “Maxillary”)

### 3.3 Study selection

Eligibility assessment was performed by screening retrieved studies’ titles. Abstracts of included titles were then obtained and screened to assess their accordance with eligibility criteria. Selected articles after titles and abstracts screening were obtained for full text assessment. If a study title or abstract didn’t provide sufficient information to make a decision, the full text was obtained and assessed. Eligibility assessment was performed by 2 reviewers. If disagreement occurred between the 2 reviewers, it was resolved by discussion and consensus.

### 3.4 Data collection process & data items

Data from the included studies was extracted in a custom-made data extraction sheet. The sheet was initially tested on few studies. Then, it was revised and applied for all included studies. The following information was extracted from different studies: Demographic data (age, date, number of cases); Participants (chief complaint, clinical findings); Imaging (tool, lesion shape); Histopathology; Treatment strategy (presurgical preparation, excision technique & approach, intraoperative bleeding, reconstruction); recurrence.

## 4 Results

**Fig. 9:**
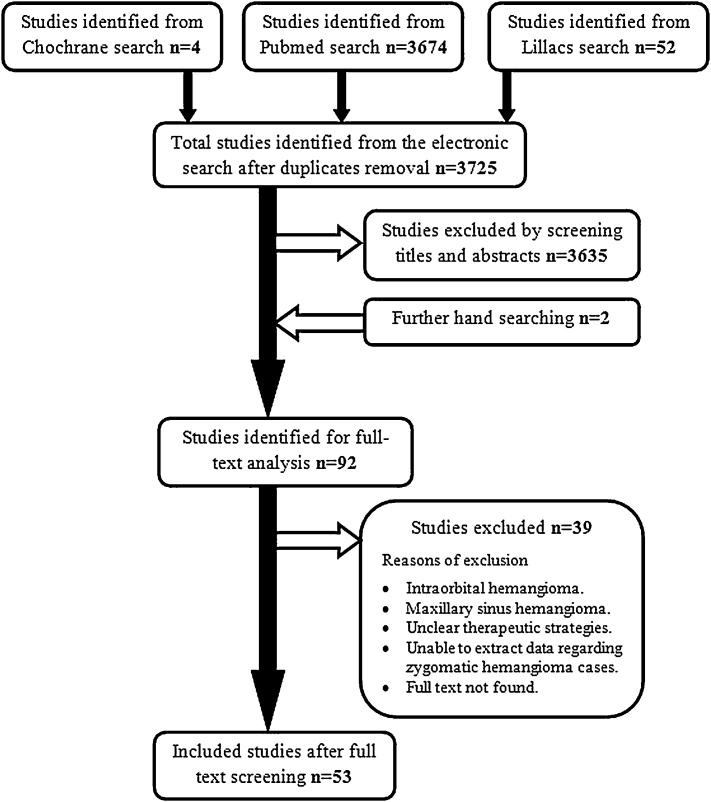
Study selection process.

**Fig. 10:**
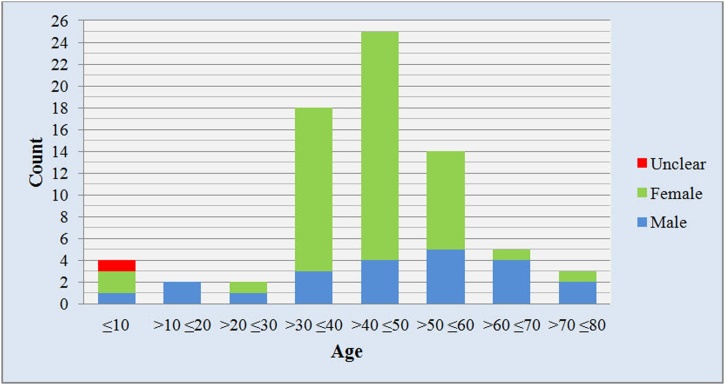
Distribution of intraosseous zygomatic hemangioma according to age.

A total of 3725 titles were identified by the electronic literature search after duplicates removal. After titles and abstracts screening and exclusion of irrelevant studies, 90 articles were selected for full text screening. 2 additional studies were found by hand searching. Full texts of 9 articles were not available, and 30 articles were excluded after full text screening. Finally, 53 articles were included in our study (Fig. 9). The included articles were case reports and case series, published from 1950 to 2018. A total of 73 cases of Zygomatic hemangioma were presented. The lesion was highly prevalent in females compared to males (2.28:1). The mean patients age was 44.1 ± 1.8 years (range 1 day-75 years). The mean age was higher in males (48.2 years) compared to females (43.2 years). The fifth decade represents the highest prevalence of the lesion (34.2 %) followed by fourth (24.7 %) and Sixth (19.2 %) decades (Fig. 10). Swelling and facial deformity was the main concern or complaint of all patients except one patient (case 43). In 11 cases (15 %), the swelling was tender on palpation. It was associated with pain in 13 cases (17.8 %); and paraesthesia in 1 case (1.4 %). Ocular findings were associated with other symptoms in 8 cases, and it was the only clinical finding in 1 case (case 43). Previous trauma was reported in 7 cases (9.6 %); and one case gives a history of previous Caldwell-Luc operation.

Plain x-rays were used as a sole imaging tool in 11 case reports; MRI was performed in 12 cases accompanied with CT, and in one case as a single imaging tool (case 59). CT scans were performed in the remaining cases. Cases showed well-defined expansile bony lesion with internal multilocular trabeculated pattern, sunburst, honey comb or Soap bubble pattern in plain x-ray and CT; intermediate T1, and high T2 signal intensity in MRI. Carotid angiography was performed in 12 cases (16.4 %). Six cases showed no pathological blood supply, while in the other cases the lesion was supplied by a prominent blood supply. Two cases were supplied from the Infraorbital, posterosuperior alveolar, anterior deep temporal, and facial arteries (cases 29, [62]); three cases from the facial, and internal maxillary arteries (cases 4, [20,61]); one case from the maxillary artery (case 16) (Table 1).

**Table 1:** Demographic, clinical, radiographic, and histopathologic findings of included cases.

Author (year)	Case	Demographic data		Clinical findings (duration) *			Etiology	Imaging		Angiography	Histopathology
		Date	Age/Sex	Swelling	Pain	Ocular findings		Tool	Shape		
Schoefield [9] (1950)	1	1949	18 mo/M	Swelling (6 mo)	N	NR	N	Plain X ray	Expanded cortical bone	NP	Hemangioma
Walker [10] (1965)	2	1961	40 Y/M	Swelling (4 Y)	Tenderness	NR	N	Plain X ray	radiotranslucent with trabeculated appearance on the tangential view	NP	Hemangioma
Walker [10] (1965)	3	1962	10 Y/F	palpable irregularity	Tenderness (6 mo)	N	T	Plain X ray	Irregularity with bony spicules radiating outward	NP	Hemangioma
Davis [11] (1974)	4	NR	47 Y/F	Swelling (2 Y)	Tenderness	NR	N	Plain x ray, Tomograms	reticulated internal pattern	tumor "blush” with prominent blood supply from left FA and IMA	Cavernous hemangioma
Brackup [12] (1980)	5	1977	46 Y/F	Swelling (2 mo)	Pain, Tender	N	N	hypocycloidal tomography; Tc-99	Honeycomb rarefaction on cut-sagittal, "Sun-ray" appearance on tangential view; increased nucleotide concentration	NP	Cavernous hemangioma
Marshak [13] (1980)	6	NR	53 Y/F	Swelling (1.5 Y)	occasionally Painfull	NR	Caldwell-Luc operation for chronic suppurative sinusitis (at 31 Y)	Plain x ray (Waters' view)	Reticulated honeycomb pattern	NP	Capillary hemangioma
Marshak [13] (1980)	7	NR	35 Y/F	Swelling (1 Y)	Pain	Globe pushed upward	N	roentgenograms	Reticulated internal pattern	NP	Capillary hemangioma
Hornblass [14] (1981)	8	1981	53 Y/F	Swelling (1.5 Y)	Pain (3 Y)	N	N	Plain x ray; CT scan	Area of rarefaction and increased radiolucency; bony thickening of the right lateral orbital wall	NP	Cavernous hemangioma
Schmidt [15] (1982)	9	1977	43 Y/F	Swelling (< 1 Y)	N	NR	T	Plain x ray	spongy appearance with multiple striations (sunburst appearance)	NP	Hemangioma
Har-el [16] (1986)	10	NR	60 Y/M	Palpable non tender mass	Pain (2 Y)	NR	N	Plain x ray (Waters' view); CT	Radiodense mass projecting into the antrum; mass originating in the zygomatic bone, with varying bony and soft tissue densities, lateral wall of the sinus thin and missing at some points	NP	Cavernous hemangioma
Har-el [17] (1987)	11	NR	43 Y/F	Swelling (4 Y)	N	NR	N	Plain x ray	irregular reticular pattern	NP	mixed cavernous/capillary bone hemangioma
Har-el [17] (1987)	12	NR	47 Y/F	Swelling (3 mo)	N	NR	N	Plain x ray	irregular pattern, upward displacement of orbital floor	NP	Cavernous hemangioma
Warman [18] (1989)	13	NR	38 Y/F	Swelling (4 month)	Pain (4 months)	NR	N	CT; MRI	Rarefied, monostotic, well-circumscribed; isointense with muscle (T1), hyperintense (T2)	NP	mixed cavernous/capillary bone hemangioma
Jeter [19] (1990)	14	NR	1 day	Swelling	–	–	–	Plain x ray	loss of cortex	NP	Cavernous hemangioma
Jeter [19] (1990)	15	NR	50 Y/F	Swelling (1 Y)	N	NR	N	Plain x ray	radiolucent lesion	no vascular malformations of the left midface	Cavernous hemangioma
Nishimura [20] (1990)	16	1962	69 Y/M	Swelling (5 Y)	N	Proptosis	N	CT; MRI	expansive soft-tissue density mass in maxillary sinus, irregularly mineralized matrix; low-signal-intensity mass (T1), high-signal-intensity mass (T2)	feeding vessel from the left maxillary artery	Cavernous hemangioma
Clauser [21] (1991)	17	NR	56/F	Swelling (4 Y)	N	NR	N	CT	NR	External carotid no pathological blood supply	Cavernous hemangioma
Clauser [21] (1991)	18	NR	35 Y/F	Swelling (1 Y)	Tender	NR	N	CT	NR	External carotid, no pathological blood supply	Cavernous hemangioma
Tang Chen [22] (1991)	19	1989	44 Y/F	Swelling (1 Y)	N	N	N	Plain x ray	radiolucent lesion	NP	Capillary hemangioma
Cuesta Gil [23] (1992)	20	NR	10 Y/F	Swelling (2.5 Y)	Pain (4 Y)	superior displacement of the eyeball	N	Plain x ray; CT	oval, radiopaque; mixed density mass	“Blush” with prominent blood supply from the FA and IMA	Cavernous hemangioma
De Ponte [24] (1995)	21	NR	60 Y/M	Swelling	N	N	NR	CT	NR	NP	Hemangioma
De Ponte [24] (1995)	22	NR	43 Y/F	Swelling	N	N	NR	CT	NR	NP	Hemangioma
Hirano [25] (1997)	23	NR	42 Y/F	Swelling (16 mo)	N	NR	N	CT	Radiolucent tumor	NP	Cavernous hemangioma
Hirano [25] (1997)	24	NR	46 Y/F	Swelling (1 Y)	N	NR	N	CT	Low density tumor	NP	Cavernous hemangioma
Pinna [26] (1997)	25	NR	56 Y/F	Swelling (4 Y)	N	NR	NR	Plain x ray; CT	“honeycomb” pattern	External carotid artery, normal	Hemangioma
Pinna [26] (1997)	26	NR	35 Y/F	Swelling (1 Y)	Tender	NR	NR	Plain x ray; CT	radiolucent lesion; rarefied area with “sunburst” pattern	External carotid artery, normal	Hemangioma
Savastano [27] (1997)	27	NR	41 Y/F	Swelling (12 Y)	Tender	NR	N	CT	Mixed density mass	NP	Hemangioma
Konior [28] (1999)	28	NR	25 Y/F	Swelling (6 mo)	N	NR	N	Plain x ray; Bone scan; CT	radiotranslucent lesion; increased radionuclide in the involved area uptake; suggestive of fibrous dysplasia	NP	Mixed hemangioma
Moore [5] (2001)	29	NR	31 Y/F	Swelling (1 Y)	N	progressive dystopia (3 mo)	N	CT; MRI	expansile, rounded, well-defined lesion, overall spokewheel appearance; intermediate T1 signal intensity and a high T2 signal intensity	Hypervascular lesion supplied by external carotid artery (IOA, PSAA, ADTA, FA)	Hemangioma
Colombo [29] (2001)	30	NR	75 Y/M	Swelling	N	N	NR	CT	bony lesion, with internal radiating trabeculations, and honeycomb pattern	NP	Cavernous hemangioma
Sary [30] (2001)	31	NR	46 Y/M	Swelling (5Y)	N	NR	NR	CT	Mass with varying bony and soft-tissue densities	NP	Cavernous hemangioma
Koybasi [31] (2003)	32	NR	33 Y/F	Swelling (2 mo)	N	NR	NR	CT	Hypointense, honeycomb-like appearance	NP	Cavernous hemangioma
Leibovitch [32] (2003)	33	NR	47 Y/F	Swelling (2 Y)	N	N	NR	CT	internal radiating trabeculations and a honey comb pattern	NP	Cavernous hemangioma
Taylan [33] (2003)	34	NR	30 Y/M	Swelling (3 mo)	N	NR	NR	CT	spongious bony appearance with lobulated contour	NP	Hemangioma
Perugini [34] (2004)	35	1989	60 Y/M	Swelling	N	N	NR	CT	well-marked, dense, expansive masses	NP	Mixed hemangioma
Perugini [34] (2004)	36	1993	43 Y/F	Swelling	N	N	NR	CT	well-marked, dense, expansive masses	NP	Cellular hemangioma
Perugini [34] (2004)	37	1995	32 Y/F	Swelling	N	N	NR	CT	well-marked, dense, expansive masses	NP	Cavernous hemangioma
Perugini [34] (2004)	38	1995	46 Y/F	Swelling	N	N	NR	CT	well-marked, dense, expansive masses	NP	Cellular hemangioma
Perugini [34] (2004)	39	1997	32 Y/M	Swelling	N	N	NR	CT	well-marked, dense, expansive masses	NP	Mixed hemangioma
Perugini [34] (2004)	40	1998	38 Y/F	Swelling	N	N	NR	CT	well-marked, dense, expansive masses	NP	Cellular hemangioma
Ramchandani [35] (2004)	41	NR	38 Y/F	Swelling (2 Y)	N	N	T (2 years previously)	Plain x ray; CT	Radiopaque mass; circumscribed mass, incidental small orbital floor fracture	NP	Cavernous hemangioma
Cheng [36] (2006)	42	NR	50 Y/F	Swelling	N	N	NR	NR	NR	NR	Cavernous hemangioma
Riveros [37] (2006)	43	NR	72 Y/F	N	N	Proptosis, mobility restriction	N	CT; MRI	mass arise from zygomatic rim	NR	Hemangioma
Zins [38] (2006)	44	NR	36 Y/F	Swelling	NR	NR	NR	CT	salt and pepper appearance	N	Cavernous hemangioma
Curtis [39] (2007)	45	NR	55 Y/ F	Swelling (2,3 mo)	N	N	N	CT	expansile radiolucency with mixed-density bone	N	Cavernous hemangioma
Gomez [40] (2008)	46	NR	35 Y/F	Swelling (3 Y)	N	N	NR	CT	Well-defined, hypodense bony lesion with reticular pattern	N	Hemangioma
Valentini [41] (2008)	47	2003	57 Y/M	Swelling (4 Y)	paresthesias	NR	N	CT	lytic lesion, involving the soft surrounding tissues, both deep and superficial, of approximately 15 mm	N	Hemangioma
Madge [42] (2009)	48	NR	49 Y/F	Swelling (1.5 Y)	Pain (1.5 Y)	N	NR	CT; MRI	isolated lesion replaced the internal marrow and enhanced with contrast	N	Cavernous hemangioma
Srinivasan [43] (2009)	49	NR	66 Y/F	Swelling (4 Y)	N	NR	N	CT	Bony mass with radiating spoke wheel pattern of trabeculae	N	venous malformation
Arribas-Garcia [44] (2010)	50	2001	42 Y/F	Swelling	N	NR	NR	CT	expansile lytic rounded mass	N	Cavernous hemangioma
Dhupar [45] (2012)	51	NR	34 Y/F	Swelling (7 Y)	Tender (7 Y)	NR	T	Plain x ray; CT	multilocular radiolucency with honeycomb appearance; mixed density mass	N	Cavernous hemangioma
Marcinow [46] (2012)	52	NR	47 Y/M	Swelling (6 mo)	Pain (6 mo)	N	N	CT	well-circumscribed mass with a ground-glass matrix	N	Cavernous hemangioma
Gupta [47] (2013)	53	NR	61 Y/M	Swelling (6 Y)	N	limited infraduction, diplopia on downgaze	NR	CT	well-defined mass, with small signal voids	N	Cavernous hemangioma
Gupta [47] (2013)	54	NR	69 Y/M	Swelling (6 mo)	Pain (6 mo)	diplopia on downward gaze	NR	CT	Round expansile mass, sunburst appearance	N	Cavernous hemangioma
Gupta [47] (2013)	55	NR	40 Y/M	Swelling (1 mo)	Pain (1 mo)	N	NR	CT	Partially destructive mottled lesion	N	Cavernous hemangioma
DeFazio [48] (2014)	56	2011	58 Y/F	Swelling (2 Y)	N	N	T	CT; MRI	well-marginated bony mass, “sun-burst’’ pattern of radiating trabeculae; high signal intensity (T2)	N	venous malformation
DeFazio [48] (2014)	57	2011	53 Y/F	Swelling	Tender	NR	N	CT; MRI	consistent with a diagnosis of intraosseous venous malformation	N	venous malformation
DeFazio [48] (2014)	58	2011	49 Y/M	Swelling (6 mo)	N	N	N	CT	mass with a trabeculated “honeycomb’’	N	venous malformation
Kaya (2014) [49]	59	NR	42 Y/F	Swelling (3 mo)	N	N	NR	MRI	well circumscribed mass	N	Cavernous hemangioma
Werdich (2014) [50]	60	NR	64 Y/M	Swelling (6 mo)	Pain (6 mo)	N	Prior injury, fibrous dysplasia	CT	oval-to-round expansile lesion “Honeycomb’’ pattern	N	venous malformation
Matsumiya [51] (2015)	61	2013	59 Y/F	Swelling (3 mo)	N	NR	NR	CT; MRI	well-defined mass, honeycomb (3D CT); intermediate (T1), high signal intensity (T2)	Bilateral ECA angiograms, markedly hypertrophied branches of the left FA and IMA	Cavernous hemangioma
Hishiyama [52] (2015)	62	NR	52 Y/M	Swelling (6 Y)	N	N	T (7 Y)	Plain x ray; CT; MRI	radiopaque mass; circumscribed mass; intermediate (T1), high signal intensity (T2)	carotid arteriogram showed a hypervascular lesion, supplied by the left ECA (IOA and PSAA, ADTA, FA)	Cavernous hemangioma
Aykan [53] (2016)	63	NR	40 Y/F	Swelling (2 Y)	N	N	NR	CT; MRI	expansile round mass, radiating fine trabecula of the lesion was giving a “spoke-wheel’’ appearance on coronal images; isointense with muscle (T1), hyperintense on fat-suppressed (T2)	N	Cavernous hemangioma
Myadam [54] (2016)	64	NR	38 Y/F	Swelling (6 mo)	N	NR	N	Plain x ray; CT; MRI	-ve; expansile bony lesion; prominent trabeculations in a radiating distribution resulting in a sunburst appearance; intermediate (T1), high signal intensity (T2)	N	Hemangioma
Powers [6] (2017)	65	2013	15 Y/M	Swelling (3 mo)	N	NR	N	CT	destructive, enhancing, expanding mass	Bilateral ECA arteriograms, normal	Epithelioid hemangioma
Bocchialini [55] (2017)	66	NR	55 Y/F	Swelling (5Y)	N	NR	N	CT	lesion causing thinning and remodeling of the cortex	N	Hemangioma
Huang [56] (2017)	67	NR	35 Y/F	Swelling (7 Y)	Pain, tender	N	NR	CT	well-defined round mass, sunburst pattern of radiating trabeculae with intact cortices	N	venous malformation
Huang [56] (2017)	68	NR	41 Y/F	Swelling (2 Y)	Tender	NR	NR	CT	sunburst pattern of radiating trabeculae with intact cortices	N	venous malformation
Huang [56] (2017)	69	NR	49 Y/F	Swelling (4 mo)	N	N	NR	CT	well-defined radiolucency with trabecular density inside	N	venous malformation
Huang [56] (2017)	70	NR	44 Y/F	Swelling (3 mo)	N	eye discomfort	NR	CT	well-defined bony eminence	N	venous malformation
Choi [57] (2018)	71	NR	73 Y/M	Swelling (1 mo)	N	NR	N	CT	honeycombed osseous lesion	N	Hemangioma
Johnson [58] (2018)	72	NR	47 Y/M	Swelling	N	NR	NR	CT	well circumscribed hyperdense mass	N	Cavernous hemangioma
Fábián [59] (2018)	73	NR	15 Y/M	Swelling (4 Y)	N	Displaced eye, diplopia, mildly limited mobility	T (4 Y ago)	CT; MRI	expanded zygomatic bone with modifications in its medullar and cortical structure	Yes	venous malformation

**ADTA** anterior deep temporal artery, **ECA** External carotid artery, **F** female**, FA** Facial artery, **IMA** Internal maxillary artery, **IOA** Infraorbital artery, **mo** Month, **M** Male, **N** No, **NP** Not performed, **NR** Not reported, **PSAA** posterosuperior alveolar arteries **T** trauma, **Y** years.

Incisional biopsy was performed in 16 cases. Excessive bleeding occurred in 5 cases during biopsy and was controlled by bone wax, surgicel, pressure packs, and/or electrocautery, in one case (case 71) bleeding continued for several days after the biopsy. Aspiration was performed in 7 cases. The aspirate was blood in six cases, while the last case result was inconvenient. In 1 case core needle biopsy was performed but the result was inconclusive.

Preoperative occlusion of external carotid artery or its branches was performed in 14 cases. Ligation (with or without division) of external carotid artery performed in 2 cases (cases 4, [14]); selective embolization performed in 6 cases (cases 16, 20, 29, 61, 62, 73); cauterization of zygomatic artery performed in 6 cases (cases 35–40). 64 studies used the terms En block excision or resection / excision with safety margin / resection to describe extensive removal of the lesion including normal tissue as a treatment option for zygomatic hemangioma. Partial resection was performed in three cases (cases 42, 68, 70), in one case recurrence occurred indicating the need for En Block excision. Two cases (cases 14, 66) were treated by curettage, and recurrence occurred in one of them. In four cases (cases 43, [50,56,57]) no treatment was performed, in two of them excision was performed latter due to the increased size of the lesion. Intraoperative bleeding was minimal in almost all cases; marked bleeding was reported in three cases (cases 5, [27,32]).

**Fig. 11:**
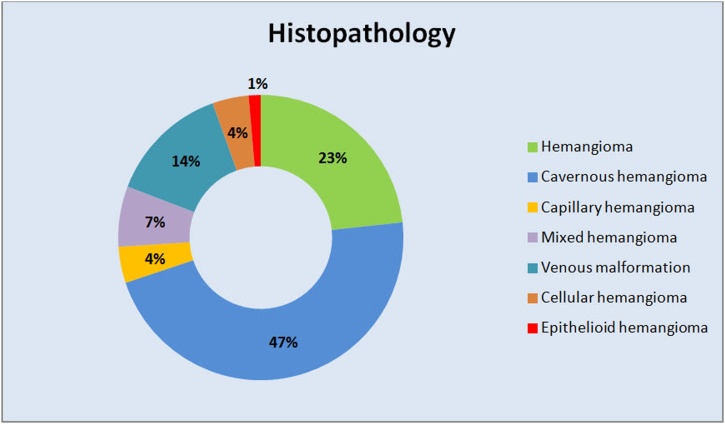
Different histopathological results of reviewed included cases.

Reconstruction was performed in 48 cases (65.8 %); in 47 cases reconstruction was performed immediately. Autogenous bone graft was used for reconstruction in 30 cases; 19 using cranial/ calverial bone graft, 5 using rib graft, 3 using iliac graft, 1 using chin graft, 1 using zygomatic buttress, and 1 using radial osteofascial graft. Computer guided surgical techniques have been utilized in five cases for planning and reconstruction of the defected zygomatic bone. One case used stereolithographic models to assist in surgical planning or for mesh pre-bending (case 41, 66), two cases reported zygomatic reconstruction using alloplastic prosthesis of methyl-methacrylate obtained from a CT-based model (case 50) or with a custom-fabricated PEEK implant (case 65) and one reported the use of patient specific implants but was not presented in details (case 73). Histopathologic analysis showed different results: 34 cavernous hemangioma, 17 hemangioma, 10 venous malformation, 5 mixed cavernous/capillary hemangioma, 3 capillary hemangioma, 3 cellular hemangioma, and 1 epithelioid hemangioma cases (Fig. 11) (Table 2).

**Table 2:** Treatment strategies and recurrence of included cases.

Author (year)	Case	Biopsy (result)	Treatment				Reconstruction Material (fixation)	Follow up	
			Preparation	Technique	Approach	Bleeding (Management)		Duration	Recurrence
Schoefield [9] (1950)	1	Ex	–	Excision	transverse incision over the malar bone	N –	NReq	NR	NR
Walker [10] (1965)	2	Ex	–	Excision with safety margin	horizontal incision along the lower lateral orbital margin	NR	NReq	NR	N
Walker [10] (1965)	3	Ex	–	Excision with safety margin	horizontal incision along the lower lateral orbital margin	NR	NReq	NR	N
Davis [11] (1974)	4	Ex	ligation ECA	Excision with safety margin	Extended infra orbital incision	Mild	Delayed (6 month), Rib graft	NR	NR
Brackup [12] (1980)	5	Ex	–	Excision	Orbital floor fracture incision	Marked	NR	NR	NR
Marshak [13] (1980)	6	Ex	–	Excision	Blepharoplasty incision lower eyelid	NR	Imm, pedicled fatty tissue from the infratemporal fossa	2 years	N
Marshak [13] (1980)	7	Ex	–	Excision	Blepharoplasty incision lower eyelid	100 ml blood loss	Imm, pedicled fatty tissue from the infratemporal fossa	20 mo	N
Hornblass [14] (1981)	8	Ex	–	Excision	Infraciliary incision	NR	NR	NR	N
Schmidt [15] (1982)	9	Ex	–	Excision	Lower blepharoplasty incision	Minimal	NR	6 mo	N
Har-el [16] (1986)	10	Ex	–	Excision	Caldwell-Luc operation	NR	NReq	4 mo	N
Har-el [17] (1987)	11	Ex	–	Excision	Infraorbital incision	Minimal	Imm, Silicone (wire)	10 Y	N
Har-el [17] (1987)	12	Ex	–	Excision	Infraorbital incision	Minimal	Imm, Silicone (wire)	NR	N
Warman [18] (1989)	13	In (Hm)	–	En bloc excision	Extended brow incision	Wih incisional biopsy (Bone wax)	Imm, free iliac bone graft	5 mo	N
Jeter [19] (1990)	14	As (blood)	Ligation, division ECA	Curettage	Weber-Fergusson incision	N	NReq	6 Y	N
Jeter [19] (1990)	15	As (blood)	–	Resection	Subciliary incision	NR	Imm, iliac crest bone graft	4 Y	N
Nishimura [20] (1990)	16	In (Hm)	Embolization MA (before biopsy), MA clipped in the pterygopalatine fossa	Excision	hemi-coronal incision with preauricular extension	N	Imm, vascularized outer-table calvarial bone flap (wires)	NR	N
Clauser [21] (1991)	17	As (blood)	–	Resection	bicoronal and right subciliary incision	N	Imm, split calvarial bone graft (microplates, screws)	NR	N
Clauser [21] (1991)	18	As (blood)	–	Resection	bicoronal and right subciliary incision	NR	Imm, split calvarial bone graft (micromesh, screws)	NR	N
Tang Chen [22] (1991)	19	Ex	–	wide local excision	coronal incision	NR	Imm, Split cranial bone graft + lateral canthopexy	6 Y	N
Cuesta Gil [23] (1992)	20	Ex	Selective embolization 24 h before surgery	resection	Coronal and infraorbital approach	Minimal	Imm, inner table of the parietal bone	3 Y	N
De Ponte [24] (1995)	21	Ex	Unclear	Excision with safety margin	Hemicoronal incision with subciliary incision	N	split cranial bone graft	NR	NR
De Ponte [24] (1995)	22	Ex	Unclear	Excision with safety margin	Hemicoronal incision with subciliary incision	N	split cranial bone graft	NR	NR
Hirano [25] (1997)	23	Ex	–	Excision	NR	NR	Imm, hydroxyapatite multiporous block	4 Y	N
Hirano [25] (1997)	24	Ex	–	Excision	NR	NR	Imm, hydroxyapatite multiporous block	8 M	N
Pinna [26] (1997)	25	As (blood)	–	Resection	NR	NR	Imm, fullthickness calvarial graft	NR	NR
Pinna [26] (1997)	26	As (blood)	–	Resection	coronal and subciliary incision	NR	Imm, partial thickness calvarial graft	NR	NR
Savastano [27] (1997)	27	Ex	–	Total resection	hemicoronal and subciliary incision	Y	Imm, autogenous calvarial flap pedicled on the temporalis fascia and muscle (miniplates)	NR	N
Konior [28] (1999)	28	In (mixed Hm)	–	Excision with 3 mm safety margin	Combined sublabial-subciliary approach	With incisional biopsy, < 50 ml with excision	Imm, outer table calvarial bone (microplates, screws)	6 mo	N
Moore [5] (2001)	29	In (Hm)	Supraselective embolization	en bloc resection	subciliary incision	With incisional biopsy (bone wax), minimal with resection	Imm, Medpor (malar bone), Ti mesh (orbital floor), Alloderm patch (sinus)	NR	NR
Colombo [29] (2001)	30	Ex	–	Excision	lateral canthotomy and cantholysis	Minimnal (bone wax)	NR	NR	NR
Sary [30] (2001)	31	Ex	–	resection	subciliary incision	NR	porous polyethylene block (zygoma), sheet (orbital floor)	2 Y	N
Koybasi [31] (2003)	32	Ex	–	complete excision	NR	less than 40 ml blood	Imm, hydroxyapatite material (Ti mesh)	1.5 Y	N
Leibovitch [32] (2003)	33	Ex	–	En block resection	temporal skin incision	Y (compressions, bone wax, diathermy)	NR	2 Y	N
Taylan [33] (2003)	34	Ex	–	Partial resection	Subciliary and gingivobuccal incisions	NR	NR	NR	N
Perugini [34] (2004)	35	Ex	cauterization of zygomatic artery	wide excision with margins of 3 mm	Hemicoronal approach	N	Imm, calvarial bone	NR	N
Perugini [34] (2004)	36	Ex	cauterization of zygomatic artery	wide excision with margins of 3 mm	Hemicoronal approach	N	Imm, calvarial bone	NR	N
Perugini [34] (2004)	37	Ex	cauterization of zygomatic artery	wide excision with margins of 3 mm	Subciliary approach	N	Imm, Medpore	NR	N
Perugini [34] (2004)	38	Ex	cauterization of zygomatic artery	wide excision with margins of 3 mm	Subciliary approach	N	Imm, Medpore	NR	N
Perugini [34] (2004)	39	Ex	cauterization of zygomatic artery	wide excision with margins of 3 mm	Subciliary approach	N	Imm, Medpore	NR	N
Perugini [34] (2004)	40	Ex	cauterization of zygomatic artery	wide excision with margins of 3 mm	Subciliary approach	N	Nreq	NR	N
Ramchandani [35] (2004)	41	In (Cv Hm)	–	Wide resection	NR	stubborn bleeding with biopsy (cautery, bone wax, pressure)	Imm. pedicled calvarial flap, stereolithographic model to assess planning	NR	NR
Cheng [36] (2006)	42	–	–	partial resection	NR	NR	Imm, calvarial bone grafts	6 mo	Yes
				then en bloc tumor excision with safety margin				NR	N
Riveros [37] (2006)	43	In (Hm)	–	Incisional biopsy, then follow up	orbitotomy	NR	NReq	NR	NR
Zins [38] (2006)	44	Ex	–	Excision with safety margin	coronal, subciliary, intraoral incisions	NR	Imm, parietal bone; full-thickness (lateral orbit), split cranial bone (floor and anterior zygoma)	6 Y	N
Curtis [39] (2007)	45	Ex	–	en-bloc resection	NR	Minor (diathermy)	corticocancellous chin bone graft (resorbable plates and screws)	NR	N
Gomez [40] (2008)	46	In	–	En bloc excision with safety margins	Sub labial, Subciliary with a Bicoronal flap	Bleeding with incisional biopsy (Surgicel); N	Imm, 3D planning, Outer calvarial bone grafts, pediculated temporoparietal galea–pericranium flap, Bichat fatty ball flap	1 Y	N
Valentini [41] (2008)	47	In (Hm)	–	Excision with safety margin	hemi-coronal & lower eye lid incision	NR	Imm, free rib, temporalis muscle	3 Y	N
Madge [42] (2009)	48	Ex	–	Excision	transconjuctival	Minimal	N	NR	NR
Srinivasan [43](2009)	49	Ex	–	Excision	Nr	N	N	2.5 Y	N
Arribas-Garcia [44] (2010)	50	In (Cv Hm)	–	Reject treatment, then complete resection after 5 Y	intraoral and the coronal approach	NR	Imm, alloplastic prosthesis of methyl-methacrylate obtained from a CT-based model, designed from 3D models of the contralateral zygoma	1 Y	N
Dhupar [45] (2012)	51	In (Cv Hm)	–	Total excision with safety margin	lateral canthotomy	Escessive bleeding with incisional biopsy (Pressure packs)	N	NR	NR
Marcinow [46] (2012)	52	Ex	–	Excision (curette, rongeurs, and a drill)	transconjuctival, sub labial incsion	NR	N	NR	N
Gupta [47] (2013)	53	Ex	–	En block excision	swinging lower eyelid flap	NR	NR	NR	NR
Gupta [47] (2013)	54	Ex	–	En block excision	swinging lower eyelid flap	NR	NR	NR	NR
Gupta [47] (2013)	55	Ex	–	Piecemeal excision	Transconjunctival approach	NR	NR	NR	NR
DeFazio [48] (2014)	56	In	–	surveillance and follow-up	–	NR	NReq	NR	NR
DeFazio [48] (2014)	57	Ex	–	Forgo treatment, then complete excision after 2 Y	NR	NR	Imm, split outer table parietal graft	NR	NR
DeFazio [48] (2014)	58	Ex	–	excision	intra-oral	NR	Imm, zygomatic buttress	NR	NR
Kaya (2014) [49]	59	Ex	–	En block resection with safety margin	subciliary	(Bone wax)	Imm, Medpor (polypropylene sutures)	28 mo	N
Werdich (2014) [50]	60	Ex	–	En bloc resection	NR	Minimal	NR	85 mo	N
Matsumiya [51] (2015)	61	In	Selective microcatheter embolization	En bloc resection	subcilial and intraoral approach	Significant with incisional biopsy; total blood loss of 500 ml	Imm, split parietal calvarial bone	3 Y	N
Hishiyama [52] (2015)	62	Ex	Supraselective embolization with poly (vinyl alcohol) particles	Complete excision with safety margin	NR	N	Imm, autogenous rib bone (Ti miniplates)	1 Y	N
Aykan [53] (2016)	63	Core needle biopsy (inconclusive)	–	Excision	NR	NR	NR	6 mo	N
Myadam [54] (2016)	64	As (inconclusive)	–	Excision	NR	Minimal	Imm, bone graft	NR	NR
Powers [6] (2017)	65	In (epithelioid Hm)	–	En block resection	transconjuctival, lateral canthotomy, intraoral	minimal (50 ml)	Imm, custom made PEEK implant using stereolithographic model	1 Y	N
Bocchialini [55] (2017)	66	In 5 Y previously (Hm)	–	curettage	transconjunctival	NR	Imm, Customized titanium grid with stereolithographic model	NR	Yes
				Excision	Transconjunctival with lateral canthotomy			18 mo	N
Huang [56] (2017)	67	Ex	–	complete excision	extended Subciliary incision	50 ml	Imm, iliac bone graft (Ti miniplates)	3 Y	N
Huang [56] (2017)	68	Ex	–	partial resection	lower eyelid incision	NR	NReq	2 Y	N
Huang [56] (2017)	69	Ex	–	aggressive curettage	NR	10 ml	NReq	7 Y	N
Huang [56] (2017)	70	Ex	–	Partial resection	lower eye lid incision	30 ml	Nreq	12 Y	N
Choi [57] (2018)	71	In (Hm)	–	Resection	subciliary incision	bleeding continued for several days after biopsy; (electrocautery)	Imm, Rib graft	10 mo	N
Johnson [58] (2018)	72	Ex	–	Resection, Preplanned bony cuts	extended Subconjunctival & intraoral incisions	NR	Imm, radial osteofascial graft, Stryker Medpor (Ti miniplates)	NR	N
Fábián [59] (2018)	73	In	superselective embolization of feeding branches from ECA + ECA isolation	Resection	Weber Fergusson Dieffenbach incision	profuse with incisional biopsy (bone wax)	Imm, patient-specific implant PSI	Y	N

**As** Aspiration**, Cv** Cavernous**, ECA** External carotid artery**, Ex** Excisional**, h** hour**, Hm** Hemangioma**, Imm** Immediate**,In** Incisional, **MA** Maxillary artery**, mo** Month, **NR** Not reported, **NP** Not performed, **N** No**, NReq** Not required.

## 5 Discussion

Intraosseos hemangioma of the zygpmatic bone represents a rare condition. Thus, surgeons often have very little experience with the diagnosis and treatment of such cases [60]. In this study, we try to integrate the available data on different treatment strategies previously used for treatment of intraosseous zygomatic hemangioma.

Although the incidence of intra-osseous zygomatic hemangioma is rare, 73 cases were described in 53 case reports. Female to male ratio was 2.28:1. Age wise, the fifth decade exhibited the highest lesion prevalence with (34.2 %) and the mean age was 44.1 ± 1.8 years. Our demographic results are consistent with figures reported in previous studies except for Matsumiya et al. as they reported a female to male ratio of 4.5:1 and this can be attributed to the smaller number of cases they review in comparison to ours [51]. Despite reporting trauma rate to be 10.9 % of the cases in our study, some authors consider trauma as the main etiological factor.

By far CT scan is the radiographic examination of choice for the intraosseous vascular lesions and this was evident in our review being used in 82 % of the reviewed cases. CT was either used in combination with plain X-ray, MRI or alone [31,36]. The most commonly described CT picture is a well-defined expansile bony lesion with internal pattern either multilocular trabeculated, sunburst, honey comb or Soap bubble pattern. These CT findings are not pathognomonic to bony hemangioma and can occur in other pathologic lesions. The MRI characteristics of a hemangioma is dependent on the size of the lesion and the signal depends on the quantity of slow-moving venous blood as well as the ratio of red marrow to converted fatty marrow present within the lesion, smaller lesions may appear bright on T1 scans while it shows low signal with large trabeculae lesions [5,42]. Unlike most bone pathology, hemangiomas showed increased signal on both T1 and T2 images [61]. In our review, Studies reported that low to intermediate signals are seen on T1- weighted images, with higher signal seen on T2. Although Angiography is a more specific examination for zygomatic haemangiomas, it was employed in only 16 % of the reported cases. It showed normal vasculature in 50 % of the cases. Incisional biopsy was done in 22 % of the cases with profuse bleeding in only 1/3 of them. Selective embolization has limited benefits as claimed by different authors [28,31].

Total tumor resection is the most successful and commonly used treatment protocol for removal of the hemangiomas alone or with safety margins. It has a proved curative effect that was maintained through a described follow up periods reaching 10 years and surprisingly marked bleeding during excision occurred in only 4 % of cases. In contrast, partial excision, curettage or no intervention are warned alternative treatment options due to a proved risk of recurrence reporting 50 % of the cases treated with curettage and 33.3 % with partial resection.

Reconstruction using autogenous bone was achieved in 66 % of the cases using calvarial graft, rib graft, iliac graft, chin graft, zygomatic buttress, and radial osteofascial. The calvarial area is the most reported common donor site may be because of its surface outline that can match the surface anatomy of the zygoma. The geometrical nature of the inferior and lateral orbital rims plus zygomatic body when reconstructed with the aforementioned donor sites need a lot of trimming and/or division in to multiple pieces to fit and reform this area to its original shape. Moreover, hazards of remote donor sites exposure and morbidity like skull vault, chest, hip bone and arms [62]. Alloplastic materials including: silicone, hydroxyl apatite, polyethylene implants (MEDPOR), methyl methacrylate and polyether ether ketone (PEEK) have been used in orbital floor and zygomatic reconstruction. Moore et al. [5] was the only case report to describe the application of 2 different alloplastic materials by using MEDPOR for the malar bone and titanium mesh for the orbital floor. Implanted materials are either shaped and fitted using free hand technique or through computer aided design and manufacture.

In our case, we decided to employ a novel method by using an autogenous ipsilateral mandibular graft from the external oblique ridge and a titanium mesh with 3D printing and stereolithographic models. Anterior border and lateral shelf of the mandibular ramus has regained the normal anatomy of the tumor area (lateral and inferior orbital rims plus lateral surface of malar bone) even with the type of bone which is mainly cortical. The use of 3D printing and stereolithographic models have improved the diagnostic tools, treatment planning and better visualization of the lesion in all dimensions. Reconstruction of the defected zygomatic bone is done through mirror imaging from the normal side and mesh pre-bending on the printed 3D models for orbital floor reconstruction.

A noteworthy diversity and debate were evident in the literature regarding nomenclature of these lesions as “hemangioma” or “malformation”. Mulliken and Glowacki published a landmark paper to resolve this confusion by establishing a criterion for histochemical, cellular and clinical distinction of two different vascular anomalies: Infantile Hemangioma and Vascular Malformations. ISSVA emphasized this terminology - which was first published in 1996 and updated in 2014 - by releasing its binary classification of the Vascular Anomalies as proliferative vascular lesions “Tumors” which include infantile hemangioma, hemangioendothelioma and angiosarcoma plus “Malformations” which may take the form of capillary, lymphatic or venous slow flow type otherwise arterial fast flow or combined [1,2]. In our review, only 14 % of the cases were histopathologically diagnosed as “venous malformation”, and the rest (86 %) were diagnosed as “hemangioma”.

Greene et al. [63] (in a letter to the editor) preferred the use of “venous malformation”. They criticize the inaccurate use of “hemangioma” to describe such intraosseous lesions. They stated (based on ISSVA binary classification that the term “hemangioma” usually refers to “infantile hemangioma”, which have not been documented to occure intraosseous, furthermore they have a different treatment strategy) [63]. On the other hand, Cheng et al. [64] (in a reply to the previous letter) replied that the term “intraosseous hemangioma” is in accordance with previous published reports [64]. Moreover, many surgeons, orthopaedics, pathologists and radiologists still name these vascular malformations as “hemangioma”. Based on our review, hemangioma is by far the most commonly used term in the literature.

## 6 Conclusions

Intraosseous zygomatic hemangioma is highly prevalent in females compared to males (2.28:1), with mean age of 44.1 ± 1.8 years. The main patient concern was swelling and facial deformity. Total tumor resection can assure no recurrence proved for over 10 years of follow-up, with minimal intraoperative bleeding occurred in most of the cases. Partial resection and curettage are associated with high recurrence rate. Computer guided surgery for resection and reconstruction facilitates the surgical procedures.

## Funding

This research did not receive any specific grant from funding agencies in the public, commercial, or not-for-profit sectors.

## Ethical approval

Exempted from ethical approval.

## Consent

Written parental informed consent was obtained from the patient for publication of this case report and accompanying images. A copy of the written consent is available for review by the Editor-in-Chief of this journal on request.

## Author contribution

Each undersigned author has made a substantial contribution to the manuscript.

Dr. Ahmed Talaat Temerek was responsible for surgical procedure, data collection, and writing of the report.

Dr. Sherif Ali was responsible for searching procedure, studies selection, data collection, data interpretation, writing of the report.

Dr. Mohamed Farid Shehab was responsible for searching procedure, studies selection, data collection, data interpretation, writing of the report.

## Registration of research studies

This is not ‘First in Man’ report.

## Guarantor

Dr. Sherif Ali, Lecturer of oral and maxillofacial surgery, faculty of dentistry, Cairo university.

## Provenance and peer review

Not commissioned, externally peer-reviewed.

## Declaration of Competing Interest

Authors have no conflict of interest.
